# A Transdisciplinary Methodology for Introducing Solar Water Disinfection to Rural Communities in Malawi—Formative Research Findings

**DOI:** 10.1002/ieam.4249

**Published:** 2020-03-20

**Authors:** Tracy Morse, Kondwani Luwe, Kingsley Lungu, Levison Chiwaula, Wapulumuka Mulwafu, Lyndon Buck, Richard Harlow, G Honor Fagan, Kevin McGuigan

**Affiliations:** ^1^ Department of Civil and Environmental Engineering University of Strathclyde Glasgow United Kingdom; ^2^ Centre for Water, Sanitation, Hygiene and Appropriate Technology Development, University of Malawi (Polytechnic) Blantyre Malawi; ^3^ Department of Environmental Health University of Malawi (Polytechnic) Blantyre Malawi; ^4^ Faculty of Social Sciences and Humanities, University of Malawi (Chancellor College) Zomba Malawi; ^5^ Department of Product Design Buckinghamshire New University London United Kingdom; ^6^ Department of Sociology and Social Science Institute (MUSSI) Maynooth University Maynooth Ireland; ^7^ Department of Physiology and Medical Physics Royal College of Surgeons Ireland Dublin Ireland

**Keywords:** Solar water disinfection, Transdisciplinary, Household water treatment, Behavior change, Codesign

## Abstract

Despite the increasing volume of evidence demonstrating the efficacy of solar water disinfection (SODIS) as a household water treatment technology, there still appear to be significant barriers to uptake in developing countries. The potential of SODIS is often treated with skepticism in terms of effective treatment, volume, and safety, and is dismissed in preference for more accepted technologies such as ceramic filters and dose chlorination. As part of WATERSPOUTT (EU H2020 688928), our study used a transdisciplinary methodology to cocreate an innovative SODIS system in rural Malawi. The formative work focused on the design of 1) an appropriate and acceptable system and 2) a context‐specific intervention delivery program using a behavior‐centered design. Initial research identified specific water needs and challenges, which were discussed along with a cocreation process with potential end users, through a series of shared dialogue workshops (SDWs). Specifications from end users outlined a desire for higher volume systems (20 L) that were “familiar” and could be manufactured locally. Development of the “SODIS bucket” was then undertaken by design experts and local manufacturers, with input from end users and subject to controlled testing to ensure efficacy and safety. Concurrent data were collated using questionnaires (*n = *777 households), water point mapping (*n = *121), water quality testing (*n = *46), and behavior change modeling (*n = *100 households). These identified specific contextual issues (hydrogeology, water access, gender roles, social capital, and socioeconomic status), and behavioral determinants (normative, ability, and self‐regulation factors) that informed the development and delivery mechanism for the implementation toolkit. *Integr Environ Assess Manag* 2020;16:871–884. © 2020 The Authors. *Integrated Environmental Assessment and Management* published by Wiley Periodicals, Inc. on behalf of Society of Environmental Toxicology & Chemistry (SETAC)

## KEY POINTS

- This research used a transdisciplinary methodology to cocreate a household water treatment system (HWTS)–based solution based on solar water disinfection (SODIS) in a population using open water sources.
- By increasing effective communication between scientists (social and applied), household users, government, and industry, the HWTS design took into consideration the specific context in terms of social, economic, physical, personal, and psychosocial barriers and opportunities for behavior change and improved health outcomes.
- Formative research informed not only the design of the HWTS but also the educational tools and intervention implementation methods, to potentially increase effective and sustained uptake of SODIS.

## INTRODUCTION

In 2016 there were an estimated 1.4 million deaths from diarrheal disease, of which 60% were attributed to inadequate water, sanitation, and hygiene, and 45% of those were specifically associated with unsafe and inadequate drinking water (Pruss‐Ustun et al. 2019). Largely preventable, diarrheal disease reduction is a primary focus of the United Nations' Sustainable Development Goal 6.1: to attain universal and equitable access to safe and affordable drinking water for all by 2030 (United Nations 2015). However, achieving this goal is a significant challenge, with 785 million people worldwide still lacking access to basic drinking water services and 144 million people still collecting drinking water directly from surface‐water sources, 51% of whom live in sub Saharan Africa (WHO and UNICEF 2019).

Although interventions at the water source have been shown to decrease diarrhea, research suggests that point of use interventions, such as household water treatment and storage (HWTS) can be even more effective (McGuigan et al. 2012). Household water treatment systems methods are mainly relevant in areas where household piped water connections are not available, the available water is subject to contamination at source or as a result of poor postcollection handling (Luzi et al. 2016). Existing HWTS technologies include boiling, filtration, chemical disinfection, coagulation and flocculation, UV‐C disinfection, and solar disinfection (SODIS). All have been recently evaluated and approved following the International Scheme to Evaluate HWTS Technologies (WHO 2014).

Current SODIS practice involves filling transparent containers, commonly polyethylene terephthalate (PET) bottles (volume up to 2 L), with biologically contaminated water with a turbidity <30 nephelometric turbidity units (NTU), and exposing them horizontally to full sunlight for 6 h, after which time the water is safe to drink (Graf et al. 2010; McGuigan et al. 2012; Luzi et al. 2016). The SODIS process inactivates microbial organisms via a combination of 1) solar UV‐B; 2) solar UV‐A radiation, oxidative activity associated with dissolved O and other endogenous components in the cells; and 3) thermal conditions during solar exposure (Castro‐Alférez et al. 2016).

Solar water disinfection has been shown to reduce childhood dysentery by as much as 45% (McGuigan et al. 2012), requiring comparatively little person effort in its use and a high value for money, while also being easy to understand and use, low cost, and sustainable. However, despite this, adoption could be described as moderate, with uptake ranging from between 9% to 90% (Rainey and Harding 2005; Tamas et al. 2009; du Preez et al. 2011). Key reasons for lack of uptake of SODIS have been reported as 1) relatively small volume (2 L) of water per bottle means that numerous bottles need to be used to meet household demand; 2) in cases of high turbidity, filtration may be needed to reduce the turbidity of water preferably to below 30 NTU for effective disinfection, therefore increasing the labor involved; 3) uncertainty about its safety, when people do not believe that water is safe and some have concerns about the possibility of harmful chemicals from PET bottles leaching into the water after long‐term exposure in the sun; 4) the long treatment time to achieve disinfection (≥6 h) when compared to other methods such as chlorination; and 5) lack of integration of behavior change programs that address not only the technical aspects but also contextual and psychosocial factors which may affect uptake and sustained use (Rainey and Harding 2005; Tamas et al. 2009; Kraemer and Mosler 2012; McGuigan et al. 2012; Mosler et al. 2013; Borde et al. 2016; Keogh et al. 2017).

For all HWTS, high levels of adherence are required if health impacts are to be realized within the household. Studies have shown that without a high level of compliance (correct, consistent, and sustained use), particularly where water quality is poor before treatment, reductions in diarrheal disease can be difficult to achieve (McGuigan et al. 2011; Brown and Clasen 2012). In order to achieve this adherence to an HWTS, including SODIS, the development and rollout of the product must consider a range of factors that may influence uptake and compliance. As highlighted by Ojomo et al. (2015), there are a number of barriers and enablers to HWTS success, all of which are context specific and can be categorized in 6 domains: user preference, integration and collaboration, standards, certifications and regulation, resource availability, market strategies, and user guidance for the product. All of these issues must be taken into consideration in the development and promotion of SODIS intervention, and this can be most effectively done through the use of a transdisciplinary method to bridge the gap between science and society. By taking all stakeholder, including manufacturers', needs and concerns into account through a combination of scientific (applied and social) exploration and societal participation, effective water treatment can potentially be improved in a sustainable and acceptable way (Tress et al. 2005; Mauser et al. 2013).

This work was based in Malawi within a large transdisciplinary research project WATERSPOUTT (EU H2020 688928) (www.waterspoutt.eu), developing solar‐enhanced water treatment technologies in 4 African countries, while also integrating a social science program structured to ensure that the gap between science and society was addressed. On the technological side, building on previous work to increase the volume of water treatable by SODIS (Keogh et al. 2015), the present study focused on the development of a combined solar filtration water treatment system that could treat up to 20 L: The concept was to increase treatment volume and combine the use of filtration technologies to potentially increase efficacy and user acceptability. On the social science side, building on previous community‐based research for sustainable safe water (Fagan et al. 2015), the present study focused on the analysis of the social, political, and economic context of water use and needs, identifying the relevant governance practices that potentially impact water resourcing, and determining the water challenges faced at household, community, regional, and national levels. The transdisciplinary element would enhance knowledge production within the team and within the communities concerned to address access to safe drinking water through social design and localized adaptation, operation, and management of the integrated solar technologies.

Water challenges are not just “technical” in Malawi but relate to structural and embedded inequalities that go to the heart of the uneven development of capitalism and a globalization that has generated greater inequality between and within countries (Arrighi et al. 2003). With a population of 17.5 million, Malawi reports 85.3% coverage for access to safe drinking water (NSO 2019). However, a major challenge is the disparity in access to safe drinking water between urban and rural environments, where 16% and 84% of the population reside, respectively (NSO 2019). Household water treatment is carried out by a minority of the rural population (31%), using methods such as chlorination (64%), boiling (28%), filtration (9%), and natural settlement (17%) (NSO 2017), with chlorination primarily occurring due to free distribution during localized outbreaks of cholera. This lack of uptake for household water treatment is potentially compounded by the lack of national or local policies that relate to HWTS (Rowe 2013), despite the Government of Malawi National Water Policy vision of “Water and sanitation for all, always” (Government of Malawi 2005). A more robust system of control for HWTS is needed, while ensuring that technologies on offer are appropriate, accessible, and affordable to the end users. For this to occur, an effective enabling environment, improved coordination, and inclusion of the private sector in the development of systems are integral (Rowe 2013).

The present formative study describes the transdisciplinary method used by WATERSPOUTT in the development of a 20‐L SODIS treatment system, which aims to address the needs of the target population. In seeking to introduce an HWTS that can be successfully taken up and rolled out, the present study took into consideration the opinions of the householders, the socioeconomics of the households as the context of the design, opportunities for local and cost‐effective manufacture, and the need for complementary and appropriate educational tools. Therefore, the present study encompassed facets of technology, social context, and psychosocial factors using the De Buck et al. (2018) theory of change as a framework. Although specific to SODIS in this case, this method could be applied to other HWTS.

## METHODOLOGY

### Transdisciplinary research

The present research was undertaken using a transdisciplinary method. In other words, at all times, the research was motivated to coproduce, with societal actors at the household level, solar technologies to address the real‐world challenge of householders' reliance on unsafe water while also advancing science. The householders involved in the research were selected on the basis that they were inclined to opt for open water sources for drinking, thereby increasing their risk of waterborne diseases. In using this method (Lang et al. 2012), the team initially sought to achieve a critical social science understanding of the historical and economic dynamics of water governance in Malawi, avoiding portraying water as a purely technical issue beyond politics and taking into consideration the colonial and neocolonial power relations (Mathur and Mulwafu 2018) from the outset. Transdisciplinarity as a methodology, which in this team involved the inclusion of critical social science, health sciences, natural sciences, design, and end users, was one that would ensure that a “technical fix” outcome would not suffice.

### Formative research

The formative research was undertaken in 4 stages from May 2017 to December 2018: 1) shared dialogue workshops (*n = *5), 2) sociospatial survey (*n = *777), 3) water point mapping and testing (*n = *46), and 4) risks, attitudes, norms, abilities, and self‐regulation (RANAS) survey (*n = *100) (Mosler 2012). The process aimed to ensure that the final SODIS prototype took into consideration the specific context in terms of social, economic, physical, personal, and psychosocial barriers and opportunities for behavior change and improved health outcomes. To facilitate, interpret, and utilize data collected at all 4 stages, a transdisciplinary team of engineers, applied scientists, social scientists, public health specialists, government extension workers, private sector manufacturers, and community members were used throughout the formative process.

### Study area

Malawi is divided into 28 districts, which are subdivided into Traditional Authorities (TAs). Each TA contains villages, which are administered by chiefs and/or village heads. There are 12 TAs within Chikwawa district. Covering an area of 4755 km^2^ (NSO 2015, 2017), the district has an estimated population of 564 684 (NSO 2019) of which 16% are under the age of 5 y. Only 7.2% of the population in this area have safely managed drinking water as defined by the World Health Organization (WHO 2017b; NSO 2019).

The present study was undertaken in 17 villages (total population, 3290) in TAs Lundu and Ngabu, which were purposefully sampled, in collaboration with the District Water Office, as areas with the poorest access to safe drinking water in the district. This population currently has limited access to safe drinking water as a result of the hydrogeology of the area that renders water unsuitable for domestic use (Monjerezi and Ngongondo 2012). Consequently, households are inclined to opt for unimproved water sources for drinking, thereby increasing their risk of waterborne diseases and their potential interest in water treatment technologies, compared to their counterparts who have access to improved water sources (e.g., boreholes). Being rural, Chikwawa is one of the districts with the lowest literacy rates (58%) with an average of 4.4 people per household (NSO 2019). Diseases such as diarrhea in children under 5 y are reported as higher in Chikwawa District (26.3%) than nationally (22%) (NSO 2017).

Households recruited in the project depended on an unimproved water source (i.e., one that by the nature of its construction does not adequately protect the source from outside contamination, in particular with fecal matter) for drinking water, had a latrine, and at least 1 child less than 34 months old at the time of recruitment, verified through birth and/or immunization records supplied by the caregiver. Physical recruitment was conducted by trained research assistants with the approval and support of community health workers, traditional leaders (village chiefs), and community volunteers. Written consent was received from all households willing to participate.

### Ethical approval

Overall European Union (EU) ethical approval was obtained from the Research Ethics Body (REC) of the Royal College of Surgeons in Ireland (RCSI). Approval was also obtained from the Ethics Committee of Maynooth University (NUIM) and from the National Health Sciences Research Committee (approval number 1823) in Malawi.

### Data collection

#### Shared dialogue workshops

The transdisciplinary methodology supporting the social design and localized adaptation in the project was defined from the outset as “shared dialogue” and often took place in explicitly planned shared dialogue workshops (SDWs) between May 2017 and December 2018, each of which used a unique methodology. The purpose of each SDW was outlined at the outset. The SDWs were central to the codesign process, and five took place over the formative design period. Reports were drawn up from the captured dialogue between anyone and everyone associated with the project, whether scientist, social scientist, community worker, householder, student, politician, or business person. The SDW designs used a range of methods, in community, commercial, and academic settings, for engaging with the transdisciplinary team, including style discussions (small groups with rapid feedback) (Dickson and Tholl 2014), focus group discussions, and standard meeting formats. In addition, scientists on visits to case‐study areas took field notes that summarized interactions, directions, and decisions as they occurred in the field as they considered their design and how these inputs affected decisions. Social scientists were on hand when scientists were in the field to 1) organize or/and attend meetings between scientists and community members or community workers, and 2) organize the facilitation of those workshops (meetings) and to take notes (transcriptions) of the conversations or dialogue that took place in that community between designers and users. The SDWs built the capacity of all to understand the social context of the new technology's use, its adaptation, or its rejection. Due to the wide range and location of project partners, it was not possible for all designers and scientists to be in attendance at SDWs, but all had access to the content through shared reports, transcripts, and documentation to inform design development (Buck et al. 2017).

#### Social–spatial survey

Conducted in 777 households in 17 villages (July 2017), a structured questionnaire was developed, based on previous questionnaires exploring community‐level water resourcing and its governance (Macri et al. 2013; Fagan et al. 2015). In this case, it was designed to collate contextual data on household characteristics, household livelihood and well‐being, access to safe water, water collection and management practices, social capital, participation in community‐based water management programs, and views on water challenges. These household data were used to provide an insight on current practices, barriers to water access and treatment, and materials currently available and used for water storage and treatment, to develop a context‐appropriate HWTS. All questions were translated into the local language (Chichewa) and pretested to ensure translations were clear and appropriate. Questionnaire responses, GPS coordinates of the household, and associated main drinking water source were then collected using KoBo Collect (KoBo Toolbox 2018) on tablets.

#### Water mapping and water quality tests

Water points were mapped (GPS coordinates) and assessed for turbidity and microbiological quality to determine their suitability for SODIS treatment and the need, if any, for prefiltration of water. A community member assigned by village leadership helped in the identification of all water points used in the village. The GPS of each water point was taken in August 2017 (dry season) and December 2017 (rainy season). Specific data on each water point was collected using a standard questionnaire in KoBo Collect (https://www.kobotoolbox.org).

Water quality tests were conducted in December 2017 to determine the turbidity (Turbidity meter‐HACH 2100Q) and microbiological quality based on most probable number (MPN) of coliforms and *Escherichia coli* (Colilert test: https://www.idexx.com/en/water/water‐productsservices/colilert/). Samples were taken directly from each water source in a sterilized container and placed in a cool box at <50 °C. Samples were delivered to the University of Malawi–Polytechnic laboratory within 3 h of sampling and processed immediately using the Colilert‐18 (IDEXX, UK) and Quantitray 2000 (IDEXX, UK) systems. Following 18 h of incubation, samples were read for MPN.

#### Risks, attitudes, norms, abilities, and self‐regulation (RANAS) study

A questionnaire based on the risk, attitudes, norms, abilities, and self‐regulation (RANAS) model (Mosler 2012) and specifically designed to determine psychosocial issues pertaining to water treatment was conducted in 100 households randomly selected from the 777 households who had participated in the sociospatial survey (March 2018). The outputs of this survey, in combination with feedback from SDWs, were used to inform the development and key messages to be used in educational materials and user guides, to address not only the technical aspects of SODIS but also the long‐term changes needed for sustained use of the product based on behavior change principles. The survey also included questions on communication channels used in the area, and social networks to inform how key messages should be delivered effectively, and through which respected community members. Questions were translated into Chichewa, pretested to ensure translations were clear and appropriate, and programmed into KoBo Collect (KoBo Toolbox 2018) for completion on tablets.

### Data processing and analysis

#### Social spatial survey

Data were downloaded from the KoBo Collect platform in .xls format, cleaned, and analyzed using Microsoft Excel Version 16. The majority of the questions were structured and precoded. Open responses were grouped and recoded before analysis. Means, modes, medians, and proportions were calculated for the different variables, and results were summarized to provide an overview of the water and household context.

#### Water mapping and water quality tests

The water point mapping questionnaires and coordinates were examined using the “view on map” function provided by the KoBo Collect toolbox software. Turbidity and Colilert test results for the different water points were entered into Microsoft Excel Version 16 worksheet and graphs prepared from the data. Results were compared to both WHO (2017a) and Malawi Bureau of Standards (MBS 2005) drinking water standards.

#### RANAS survey

Data processing and analysis followed the RANAS method (Contzen and Mosler 2015). Participants were asked questions that followed a rating scale of 1 to 5, addressing different behavioral factors. In the study, “doers” were those that treated water ≥75% of the time and “non‐doers” less than 75% of the time. The data were analyzed using SPSS version 25 (IBM Corp 2017). An ANOVA mean comparison analysis to determine the differences between doer and non‐doer for the water treatment behavior was done. Behavioral factors that were noted to be significant after ANOVA calculation were further analyzed (i.e., any factor at *P* < 0.05 using ANOVA) with effect size, *d*, where Cohen's *d* values mean small for those ≤0.20, medium for those ≤0.50, and large for those ≥0.80.

## RESULTS

In order to develop and trial the prototype, it was extremely important to understand the context in which these HWTS would be used, and the social, economic, and cultural barriers that may prevent its uptake and use.

### Demographics

Seven hundred and seventy seven households were interviewed in the sociospatial survey, of which 80% were from TA Lundu and 20% from TA Ngabu. Demographics of households are summarized in Table 1. Household composition was commensurate with national statistics: majority of households were married couples headed by males, households had low levels of education, and were either subsistence farmers or farm workers. Household income was well below the World Bank extreme poverty line of US$1.90 per day. As such, any HWTS would have to consider the need for a low‐cost technology to appeal to the target population.

**Table 1:** Study participant demographics (*n = *777)

Attribute	Variable	Percentage or mean
Mean age of respondent		30 y
Gender of respondent	Males	22%
	Females	78%
Gender of household head	Males	89%
	Females	11%
Marital status of household head	Married	90%
	Divorced	6%
	Widow	3%
	Widower	1%
	Single	1%
Education of household head	Primary education	64%
	Secondary education	25%
	No schooling	11%
	Higher education and above	1%
Main household income source	Sale of labor	44%
	Crop farming	30%
	Business	13%
	Salaried worker	11%
	Mixed farming	2%
	Livestock	1%
Mean household monthly earnings		US$22

### Household water management and gender‐related issues

Fifty‐four percent of the households indicated that they had treated their drinking water at some time, of which 45% had used chlorine and 9% had used boiling. Preference for chlorination was due to its ease of use and less time to achieve treatment, as well as free distribution during localized outbreaks of cholera. Lack of firewood was the main barrier to boiling as a treatment method. Ninety‐six percent of the households had never heard of SODIS. Of the 4% that had heard of SODIS, only 39% had seen SODIS being used before, and 35% had used SODIS before but stopped and opted for other technologies due to uncertainty about its efficacy. As such, any HWTS implementation would need to both encourage water treatment as a whole and successfully promote the use of SODIS over other more traditional water treatment methods.

Women aged between 15 and 45 y were primarily responsible for water collection (98%), storage (97%), and treatment (89%), compared to their male counterparts: water collection (10%), storage (7%), and treatment (5%). Nevertheless, men were reported to be in control of water‐related financial contributions (74%) and decision making (68%) at the household level.

Women (15–45 y) collected water 3 times a day on average, and most of them (83%) used walking or head load as a mode of transport. The participants indicated they used 20‐L plastic jerry cans (82%) and 20‐L buckets (58%) to collect water and stored it in plastic jerry cans (48%) and buckets (58%). The few men collecting water had access to piped water in the adjacent sugar estate where they were employed and used bicycles as a mode of transport and water carrying. As such, 20‐L buckets were already a familiar and acceptable water collection storage container, and priority on use should target women and men to ensure the household is committed to use, both in practice and financially.

Seventy‐four percent of households had a water source less than 500 m from the household, taking an average of 56 min per return trip. Those with water sources between 500 m to ≥1 km away from their home took an average of 79 min per return trip, with those more than 2 km away taking 96 min. The main reasons for using these water sources was that it was the only water source available (53%), was closer to the household (26%), had water that was not salty (19%), or was a permanent and reliable source of water (17%).

In terms of problems faced during water collection, respondents indicated contaminated water at the source (39.6%), distance from water source to home (35.1%), congestion (30.1%), and crocodile attacks at the water point (14%). Therefore participants recognized the dangers and risks of consuming these unimproved water supplies but saw these are their only reliable sources.

### Water‐related social capital and conflicts

Of the 777 respondents, 50% said they trusted the people in their village and 33% did not, with the remainder indicating mixed levels of trust. In terms of social support, 40% said they could rely on people in the village to come to their aid when in need and 53% said they could not, with the remainder stating it was dependent on the person. Ninety‐seven percent of the households said that they had previously participated in community development activities by providing labor (81%) and finances (13%). However, developmental activities related to water were low (18%), with the majority being related to nonwater issues (54%) such as schools. This low community participation in water related activities demonstrates a limitation in the social capital of the population, with a large proportion of the population feeling unsupported. This could create issues in terms of building social norms for HWTS and for the safety and security of HWTS when left unattended at households.

Water conflicts centered around water shortage (43%) and congestion (9%), which resulted primarily in verbal (78%) rather than physical (22%) fighting, the majority of which was resolved by community members themselves (80%) but in some cases required intervention from the water committee or traditional leadership (18%).

### Water point mapping and water quality tests

Water points were mapped in August and December 2017 (Table 2). Interestingly, there were more water points in August (*n = *75) in the dry season than in December (*n = *46) in the rainy season. Because a number of the water points in August were located within dry river beds or on the banks of flowing rivers, these additional sources were abandoned in December due to rivers being in full spate, flooding of river banks, and increased turbidity due to runoff.

**Table 2:** Water points in August and December 2017

Type of water source	August (*n* = 75) dry season: nr (%)	December (*n* = 46) wet season: nr (%)
Unimproved	54 (72%)	30 (65%)
Unprotected dug well	26 (35%)	10 (22%)
River/dam/lake/pond/stream	13 (17%)	20 (43%)
Canal/irrigation channel	15 (20%)	0 (0%)
Improved	21 (28%)	16 (35%)
Borehole/deep well	15 (20%)	10 (22%)
Tap (public)	3 (4%)	1 (2%)
Protected dug well	3 (4%)	5 (11%)

**Figure 1:**
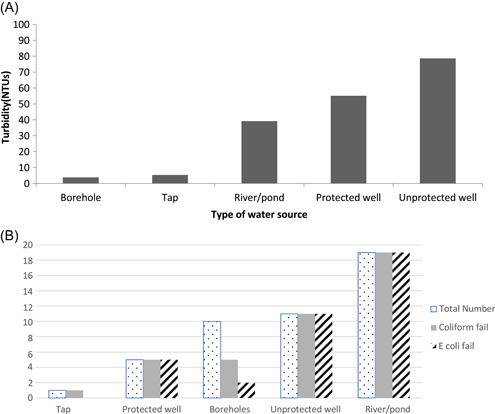
Results of water testing at source: Average turbidity of water from water points in December 2017 (**A**); number of water samples testing positive for coliforms and *Escherichia coli* in December 2017 (**B**). MPN = most probable number.

Water testing was carried out on all water points in the rainy season only (*n = *46), to be indicative of the poorest quality water due to the heavy rains. Due to their source being improved groundwater and treated water, the average turbidity of boreholes and taps was within WHO standards of <5 NTU although they were found to be contaminated at source with coliforms (tap and borehole) and *E. coli* (borehole), thereby failing microbiologically (WHO 2017a) (Figure 1). All unimproved water sources, wells (*n = *15) and rivers or ponds (*n = *20), were found to have an average turbidity higher than the required standard, and in the majority of cases higher than the 30 NTU recommended at the upper limit for effective SODIS. All of these sources, which were the primary sources of drinking water, were also contaminated with coliforms and *E. coli* in excess of WHO and MBS drinking water guidelines (MBS 2005; WHO 2017a) (Figure 1), reinforcing the need for water treatment before consumption. However, if SODIS is to be used effectively, consideration must be given to the reduction of turbidity before treatment. Although results were for the rainy season only, the consistent use of unimproved water sources all year round indicates the need for consistent use of household water treatment in this population.

### Behavioral factors influencing water treatment

Risks, attitudes, norms, abilities, and self‐regulation (RANAS) behavioral factors were tested for doers (those who treated water) and non‐doers (those who did not treat water). Following our set criterion, 71 respondents were classified as non‐doers because they reportedly did not treat water for equal to or more than 75% of the time, and 29 were doers because they treated water for more than 75% of the time. Analysis revealed that attitudes, norms, abilities, and self‐regulation were the most significant behavioral factors to be considered for inclusion when considering appropriate behavior change techniques (BCTs) to encourage SODIS uptake and use (Table 3).

**Table 3:** Significant behavioral factors identified in the doer and non‐doer analysis^a^

Factor group	Behavioral factor	Doers M(SD)	Non‐doers M(SD)	Cohen's *d*
Attitude	Like taste	2.62 (1.70)	3.28 (1.76)	0.38
Norms	Others' behavior community	3.41 (1.15)	2.63 (0.90)	0.77
	Others' behavior relatives	3.86 (1.16)	3.44 (1.40)	0.33
	Others' approval important	4.34 (0.90)	4.61 (0.77)	0.32
	Leadership promotion	3.90 (1.26)	4.42 (0.94)	0.47
Ability factors	Confidence in continuation	4.41 (1.01)	3.70 (1.34)	0.59
Self‐regulation factors	Attention to behavior	4.44 (1.95)	3.69 (1.40)	0.64

M = median.

^a^ *N* = 100; Doers = 29; Non‐doers = 71.

Subsequently, the associated BCTs were identified using the catalogue as provided by the RANAS model (Contzen and Mosler 2015) (Table 4). These BCTs were then evaluated and discussed in the context of findings from the first 4 SDWs. Methods of delivery and content were agreed through a creative workshop attended by members of the transdisciplinary team and supported by behavior change communication experts. These took into consideration the most common and preferred communication channels identified by respondents: health talks (93%) and songs and dramas (37%). The RANAS results indicated that knowledge was already high in relation to the associated risks of consuming unsafe drinking water, but there were still key misunderstandings in terms of “clean” and “contaminated” water. It also examined the aspirations of the participants as they visualized future successes on which drinking safe drinking water could be pinned. Therefore the design team focused the use of visual prompts to demonstrate bacterial transmission (e.g., use of paint to show how bacteria can move from hands to water) (disgust motives) and potential positive outcomes of drinking safe water for children through visualization of future successes (e.g., child graduating from secondary school) (nurture motives). The method of delivery through groups also sought to use traditional leaders as role models, in keeping with the identified desire for others' approval, and strengthening social networks and potential for collective efficacy (affiliation motives). Overall, these behavioral determinants were embedded in both the educational tools and the implementation guidance to support and encourage SODIS use.

**Table 4:** Behavior change techniques identified by RANAS for inclusion in educational materials and to be undertaken over 3 community meetings

Behavioral factor	Behavior change technique	Activities included in educational tools for community meetings
Attitude factors		
Feelings Likelihood of choosing raw water over treated based on taste	Describe feelings about performing and consequences of the behavior: Present the performance and the consequences of a healthy behavior as pleasant and joyful and its omission as an unhealthy behavior which is unpleasant and aversive.	Paint game demonstrating the spread of bacteria (Meeting 1). Feces in water prompt demonstrating the contamination of water from open defecation and animals (Meeting 1). Showcase of Cholera story video (in Chichewa) (Meeting 1). SODIS family drama reiterating previous messages (Meetings 2 and 3).
Norms		
Others' behavior Community Relatives Others' approval Important others Leadership promotion	Inform about others' behavior: Point out that a desired behavior is already adopted by others. Prompt public commitment: Let people commit to a favorable behavior and make their commitment public, thus showing to others that there are people who perform the behavior. Inform about others' approval or disapproval: Point out that it is important others support the desired behavior or disapprove the unhealthy behavior.	Composition and performance of songs promoting SODIS use (Meeting 2). Users provide testimonies of successes and challenges in SODIS use, and as a group discuss how to support and address these (Meetings 2 and 3). Village heads men and women participate in SODIS use, publicly advocating for its use and showing use at their homes (Meetings 1, 2, and 3). Congratulate and celebrate successful use of the SODIS systems in participating households (Meetings 2 and 3).
Ability factors		
Confidence in continuation Confidence to continuously treat water despite money problems	Reattribute past successes and failures: Prompt participants to attribute failures to a temporary lack of skill or adverse circumstances instead of to his or her deficiency and successes as personal achievements.	Users provide testimonies of successes and challenges in SODIS use, and as a group discuss how to support and address these (Meetings 2 and 3). SODIS champion rewarding ceremonies: Certificates and soap to those doing well (Meetings 2 and 3). Environmental prompts: Making a designated area for SODIS treatment (SODIS stands) (Meeting 1).
Self‐regulation factors		
Action control Attention paid to treating water	Prompt (self)monitoring of behavior: Invite participants to (self)monitor their behavior by means of recording it (e.g., frequency). Provide feedback on performance: Give participants feedback on their behavior performance. Highlight discrepancy between set goal and actual behavior: Invite the participant to regularly evaluate the actual behavior performance (e.g., correctness, frequency and duration) in relation to the set behavioral goal.	SODIS champion rewarding ceremonies: Certificates and soap to those doing well (Meetings 2 and 3). Users provide testimonies of successes and challenges in SODIS use, and as a group discuss how to support and address these (Meetings 2 and 3).

RANAS = risks, attitudes, norms, abilities, and self‐regulation; SODIS = solar water disinfection.

### Community codesign

Five SDWs were held among community members, government workers, designers, scientists (social and applied), and manufacturers between May 2017 and December 2018. These discussions were exploratory and open ended to support insights to water access and treatment issues in the population, and to allow end‐user inputs into the development and prototyping of the SODIS technology, supporting materials, and implementation methods. The design process was initially conceived to produce a combined ceramic and SODIS system, to reduce the turbidity of pretreatment water, and to increase the efficacy of the SODIS process. The controlled testing of prototype designs has been described elsewhere (Polo‐Lopez et al. 2019).

Initial design steps complemented and reinforced findings from the sociospatial survey and water testing, as described in the following 4 sections.

#### May 2017

**Figure 2:**
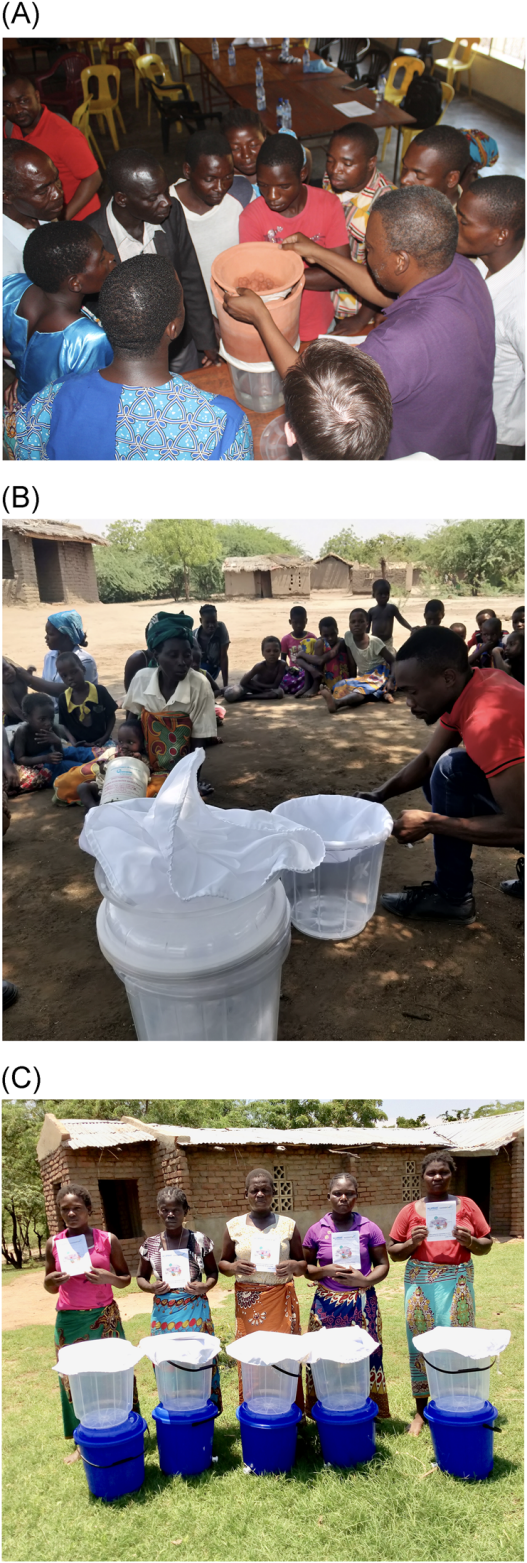
Community dialogue to assess final prototype design: Demonstrating first iteration of combined system (May 2017) (**A**); demonstrating alternative cloth filter pretreatment (September 2018) (**B**); participants for Trial of Improved Practices (TIPs; December 2018) (**C**).

Community members and extension workers (*n = *43), social scientists (*n = *4), applied scientists (*n = *3), public health researchers (*n = *3), and designers (*n = *1) participated in a series of focus group discussion sessions that explored water governance, water facilities, gender and water, and technology. These were then followed by a hands‐on interactive session with technology options to gain user perspectives on ceramic filtration, SODIS, and the first iteration of a combined filtration and SODIS system (Figure 2A). All issues raised within these sessions were noted by hand, fed back verbally to all participants for validation, and consolidated into a report. This session provided insight to community priorities for water treatment, barriers to use and perceptions of proposed technologies, and familiarity with products. Discussions validated the findings of the sociospatial survey in terms of water governance, gender and conflict, technologies, and facilities.

Community members expressed frustration with organizations who had previously attempted to provide safe drinking water in the area: “NGOs and organizations come to help, but they either just drill boreholes and leave without follow‐up, or they do not drill at all as they realize there are no solutions for the area with standard boreholes due to the salty water”—Male Community Member.

They also highlighted specific examples the difficult task of changing gender norms around household water management: “Men use culture as an excuse for why women must collect water, although men are more likely to contribute to water collection if it is far away and they can use a bicycle for collection”—Water Monitoring Assistant. “We can ask our husbands to buy chlorine, but they may refuse saying that the water has always been fine until now”—Female Community Member.

Findings were used by designers in the continued development of prototypes, particularly the desire to have a product which was at least 20 L in volume, familiar, robust, required minimal time to prepare (less than 2 h to filter), and could be locally manufactured to minimize cost. The buckets and clay filters shown within SDWs were similar to those used as water storage containers within the villages and were therefore widely accepted. They also highlighted issues to be considered during implementation, such as male inclusion in rollout, highlighting cost and time benefits compared to other treatment methods, and consideration of distance for water collection.

#### October 2017

Community members and extension workers (*n = *27), national ethics coordinators (*n = *1), and academics and research assistants (*n = *19) participated in a world café event to explore issues of trust around community health research.

Participants expressed concerns about the way that some researchers had previously come into communities without using appropriate structures and communication channels, and without respecting local cultural customs: “Researchers do not explain what they want and what they will do with the information they collect. This makes it difficult for us to appreciate the importance of their research. Poor communication means we don't participate fully, and in the worst‐case scenario, we might even give false information”—Village Chief.

During data collection, participants spoke about a lack of understanding of study eligibility criteria, misconceptions around taking human samples, and lack of communication about other research methods. Participants felt it was particularly important at the end of data collection that they receive feedback on what researchers found. This insightful meeting provided clear guidelines and expectations from participants and government officials on how the intervention should proceed once prototypes were finalized, and highlighted a high level of acceptance for the codesign approach being used to overcome research concerns.

#### June 2018

Following the controlled testing of a series of prototype iterations, meetings were held with manufacturers of ceramics, polypropylene buckets, and tailored cloth filters to explore opportunities for the local manufacture of prototypes. This process aimed to explore local manufacture costs and logistics to minimize prototype costs and maximize sustainability of production. Findings showed that local manufacture of the buckets could be easily achieved at the cost of $3 per container, commensurate with the cost of buckets of the same size for normal household use. However, the manufacture of the ceramic filter to reduce turbidity was untenable in terms of local production costs ($50) and therefore tailored cloth filters were explored as an alternative ($3.50).

#### September 2018

Following controlled testing of ceramic filter options, and taking into consideration local manufacturing costs and the turbidity of the source water (Table 2), it was decided that the ceramic filtration was not the most effective solution for the combined system, but rather a simple cloth filter. To evaluate acceptability and efficacy of the ceramic filter versus cloth filter, a community meeting was held with female household members (*n = *7), extension workers (*n = *3), a public health researcher, and the prototype designer (Figure 2B). Women indicated acceptance and familiarity with the cloth filter and suggested simplifications to the design, which were addressed in the final iteration. They also provided a series of recommendations on how they could realistically manage the treatment system, clean the system, and ensure there was always adequate treated water in the household. Lastly, the time commitments associated with community meetings and education were discussed. Participants indicated that meetings were welcome but should be minimized to reduce time burden on the household members. This information was taken into consideration during the development of the educational materials. Field testing of the filters also showed adequate turbidity reduction to facilitate SODIS (Supplemental Data Appendix 1).

### Moving toward trial

**Figure 3:**
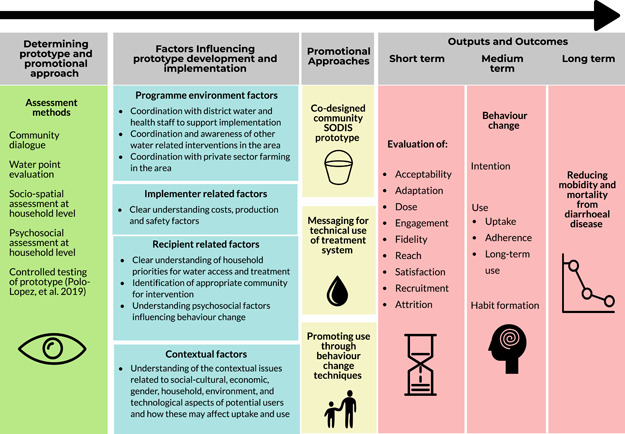
Transdisciplinary process of developing solar water disinfection (SODIS) prototype for community water treatment intervention (based on De Buck et al. 2018).

The triangulation of findings from data collection and community dialogue to this stage showed that the development and implementation of the codesigned SODIS system required 3 elements (Figure 3):

1) A codesigned prototype that took into consideration the community's requirements and was effective in water treatment. This was achieved through the SDW process and controlled testing reported elsewhere (Buck et al. 2017; Polo‐Lopez et al. 2019).
2) Effective communication of how the technology should be used to achieve safe drinking water.
3) Embedded program to support effective behavior change communication, including the use of nurture, affiliation and disgust motives, and BCTs identified by the RANAS results to stimulate sustained change.

The prototype design and testing were completed taking into consideration the inputs of the shared dialogue workshops (Buck et al. 2017; Polo‐Lopez et al. 2019). To support the rollout of the tested prototype, the data from all stages were used with a design team to develop educational tools. These tools were developed in 2 parts: 1) user manual to provide technical information needed to ensure water was safely treated (SODIS method) and 2) facilitator manual to provide guidance and support for extension workers to roll out the water treatment system in user communities encompassing technical training with BCTs identified from the RANAS model (Table 4). These activities particularly addressed issues of how users “feel” after treating water (attitudes), the need for others to adopt the behavior and the need for approval of others (norms), user confidence to be able to continuously treat water (ability), and users' ability to pay attention to using the HWTS effectively each time (self‐regulation). This program was designed to encompass 3 community visits to support and promote SODIS use, with the activities being interactive to promote and encourage attendance of household members. The user manual took into consideration the low literacy levels of the target population, focusing on a pictorial depiction of prototype use with minimal text translated to the local language (Chichewa).

The final SDW for the design stage was undertaken as a Trial of Improved Practices (TIPs) (Manoff Group 2005) in December 2018 (Figure 2C). The trial included 13 households that were provided with the final prototypes and the user manual and were trained using the facilitator guidance. The prototypes were used for 2 wk, and feedback on water treatment systems and educational tools was solicited through a focus group discussion. Findings resulted in minor changes to educational tools to make them more realistic in the time allocated for implementation, and feedback supported the decision to provide 2 systems to each household to allow up to 40 L (i.e., two 20‐L containers) to be treated at a time.

## DISCUSSION

Findings from the present study show that provision of potable water to communities in areas with poor water access such as TA Lundu, Chikwawa district, remains a serious challenge for both policy makers and technology developers. Although this makes them effective targets of an HWTS, low incomes (<$22 per month) and the long‐term constant water‐related poverty in these areas play a significant role in the decisions made around water access and water treatment. Not only do issues of water access need to be considered, but deep‐seated social and cultural attitudes to water source preferences, gender roles, and risks associated with drinking contaminated water need to be overcome if HWTS interventions such as SODIS are to succeed. In order to address these concerns, the present study sought to develop an in‐depth understanding of the context in which a SODIS prototype would be deployed. The transdisciplinary method used to approach this challenge used not only the skills of applied and social scientists, but also the knowledge and inputs of potential beneficiaries and manufacturers of the prototype to ensure we bridged the gap between science and society. The process described here is a reflection of the formative stage of prototype and intervention development. However, in order to achieve the transdisciplinary science proposed, this dialogue must be continued as households use the prototype over a longer period of time, with concurrent engagement with policy and programming stakeholders.

As found elsewhere in the region (Burt et al. 2017), water collection and treatment are highly gendered activities, and HWTS must not add to the already high burden of work placed on women in the household. However, financial support for HWTS lies either with male household heads or as a joint decision of couples, and therefore any promotion of a system must be cognizant of both male and female perceptions and financial priorities. With the average income of households in the present study being well below the poverty line, it is essential that any solution must reflect their ability to pay. Previous studies have found a higher willingness to pay in households who have more turbid water, as is the case here (Burt et al. 2017). However, the value placed on HTWS is often less than the commercial cost, leading to the need for subsidies and therefore a lack of sustained use. The design of a simple SODIS bucket reflects a container that is already familiar to households, can be manufactured locally for the same price as households already pay for containers, and is therefore potentially more accessible, affordable, and acceptable (social norm) as a HWTS, a need highlighted by previous studies (Polyzou et al. 2011; Brown and Clasen 2012; Ojomo et al. 2015). The bucket is also a one‐off payment for an HWTS, as opposed to the commercially available Waterguard (chlorine) treatments, which require a regular investment from household income to maintain safe drinking water or other filter systems that currently retail at approximately $20 per unit.

Due to the high turbidity of source water, there was a need to implement a pretreatment filter to increase the efficacy of SODIS. Although studies have shown that SODIS can achieve safe drinking water with a turbidity of up to 200 NTU, overnight regrowth of pathogens may be of concern, and therefore achieving turbidity of <30 NTU should be maintained as the ideal (Keogh et al. 2017). Although the use of ceramic filters would be best to achieve this given the turbidity levels found in the study area, the local manufacture of these as alternatives was found to be untenable because village‐level pot filters filtered too slowly (several hours) and commercially manufactured alternative filter designs were too expensive. As such, the use of cloths, which are familiar to the community and can be made locally, were found to provide the necessary reduction in turbidity levels to allow effective SODIS to take place. Alternative methods could also be explored in this population for reducing turbidity such as *Moringa oleifera*, which is grown locally. However, the time taken to prepare powder and await settlement (Keogh et al. 2017) was considered an additional burden on women's time in the household, which the HWTS was trying to minimize, and therefore was not considered in this case.

By using a transdisciplinary team to achieve codesign and development of the SODIS, the present study has aimed to overcome many of the barriers identified to household water treatment and specifically SODIS uptake through both the final prototype design and the development of supporting materials that are context appropriate and address specific behavioral determinants (Rainey and Harding 2005; Tamas et al. 2009; Kraemer and Mosler 2012; McGuigan et al. 2012; Mosler et al. 2013; Ojomo et al. 2015; Borde et al. 2016; Keogh et al. 2017). Through the use of the theory of change developed by De Buck et al. (2018), we collated data and held community discussions that examined the context fully and informed both the actual HWTS design and appropriate promotional techniques to use (Mosler 2012). The resulting prototype and user materials (with embedded BCTs) therefore aim to overcome the limitations of previous studies by understanding the preferences, choices, and aspirations of the at‐risk populations and by providing instructions that overcome potential incorrect adoption (Albert et al. 2010; Ojomo et al. 2015). The BCTs implemented through community engagement are specifically designed to promote water treatment in households through motives and the development of social norms. As a result, if successful, this engagement should support the development of a demand for the product, which local manufacturers can then fulfill.

In terms of implementation, we have tried to find a balance between the need for regular household contact (Tamas et al. 2009; Brown and Clasen 2012; Mosler et al. 2013) with the realistic abilities of government extension workers in the area to measure opportunities for scale‐up after trial. To support this lighter touch method, the tools recommend the use of community leadership (traditional, religious, government workers, etc.) and volunteers as change agents who will be in place for the long term and can integrate the promotion of the SODIS system with other water, sanitation, and hygiene interventions (Ojomo et al. 2015). A similar approach can be used for other HWTS.

The TIPs process resulted in minor changes in educational materials and helped to inform how households would like to receive the SODIS system. Although the present field test was short in duration and small in size, the use of TIPs has been shown to provide valuable evidence for larger scale rollout and programming in multiple health‐related sectors, and it is anticipated that it will increase the acceptance and use of the SODIS system in the subsequent health impact study for this HWTS (Harvey et al. 2013; USAID 2014; Shivalli et al. 2015).

The transdisciplinary development and evaluation of this prototype is ongoing as the field trials with households will now be undertaken on a larger scale, with continued engagement with manufacturers and policy makers for future deployment if found to be successful.

## SUPPLEMENTAL DATA

**Appendix 1.** Field test results for cloth filters and turbidity reduction.

## Supporting information

This article contains online‐only Supplemental Data.

Supporting information.

## Data Availability

Data presented in this manuscript are available from https://doi.org/10.5281/zenodo.1478547.
