# Supplementary figures and images for: A Transdisciplinary Methodology for Introducing Solar Water Disinfection to Rural Communities in Malawi—Formative Research Findings
Source: Integr Environ Assess Manag. 2020 Mar 20;16(6):871–84. doi: 10.1002/ieam.4249 (PMC7687190; doi:10.1002/ieam.4249)

Appendix 1: Field test results for cloth filters and turbidity reduction


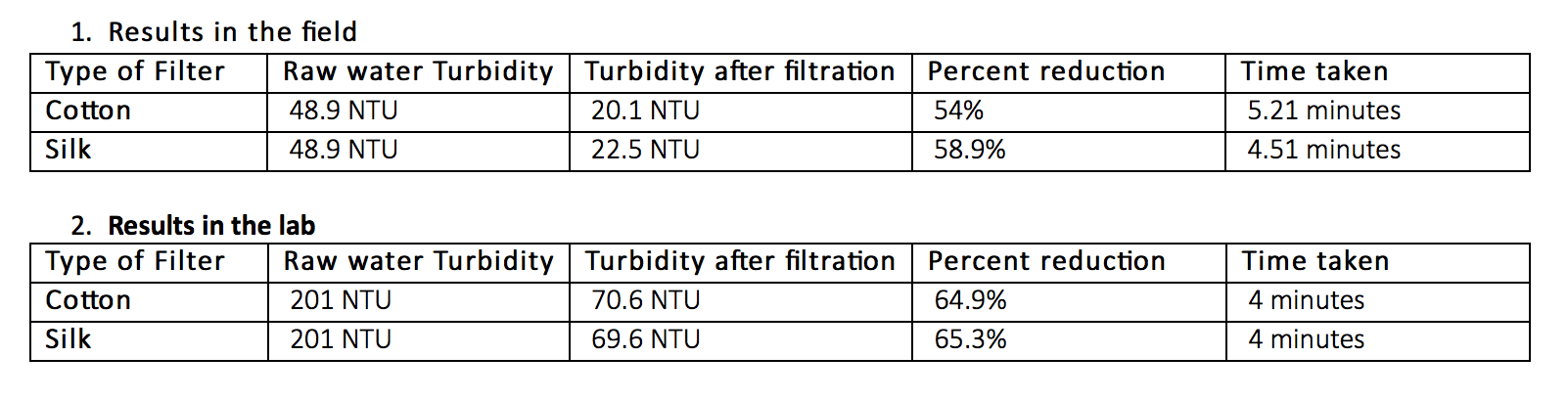

Supplement: Supplementary file 1 — Supporting information. [file IEAM-16-871-s001.docx]
